# Pseudorabies virus tegument protein pUL49 antagonizes cyclic GMP-AMP synthase through phase separation to promote viral replication

**DOI:** 10.3389/fmicb.2026.1873742

**Published:** 2026-07-02

**Authors:** Lipeng Gan, Yuan Gao, Lina Ma, Yutong Zhang, Shiyi Chai, Huiying Li, Mengting Zhang, Xinya Yang, Qingcui Xiao, Yinli Xie, Longwei Zhao, Peng Sun, Zhiyong Li

**Affiliations:** 1 School of Basic Medical Sciences, Wenzhou Medical University, Wenzhou, Zhejiang, China; 2 Cixi Biomedical Research Institute, Wenzhou Medical University, Zhejiang, China; 3 The Second School of Clinical Medicine, Wenzhou Medical University, Wenzhou, Zhejiang, China

**Keywords:** cGAS, phase separation, pseudorabies virus, pUL49, viral replication

## Abstract

Pseudorabies virus (PRV) is an economically important swine alphaherpesvirus with zoonotic potential. The mechanisms underlying PRV innate immune evasion remain poorly understood. Here, we identify the PRV tegument protein pUL49 as a critical viral factor that undergoes phase separation to antagonize cyclic GMP-AMP synthase (cGAS)-mediated antiviral signaling. pUL49 exhibits the strongest predicted phase-separation propensity and forms dynamic, 1,6-hexanediol (1,6-HEX)-sensitive condensates in cells. It colocalizes and interacts with cGAS, and its overexpression attenuates poly(dA:dT)-induced IFN-β signaling. Moreover, cGAS deficiency facilitates PRV replication, while pUL49 knockdown suppresses viral replication. These findings further elucidate our mechanistic understanding of PRV pUL49-mediated immune evasion and provide a potential target for developing antiviral strategies against PRV.

## 1 Introduction

Pseudorabies virus (PRV), the causative agent of Aujeszky’s disease, is an economically important swine alphaherpesvirus that causes fatal neurological diseases, resulting in substantial losses to the global swine industry (Bo and Li, 2022). Recently, emerging PRV variants have posed the challenges to disease control because of the reduced protection by conventional vaccines and potential to cross the species barrier. Increasing numbers of human cases associated with PRV infection, including encephalitis and endophthalmitis, have raised concern over its zoonotic potential and broader public health relevance (Huang et al., 2022; Wang et al., 2026; Zhang et al., 2023). These variants highlight the urgent need to elucidate the molecular basis of PRV pathogenesis and host immune evasion (Pomeranz et al., 2005; Sun et al., 2016).

The host innate immune system constitutes the first line of defense against viral invasion. Cyclic GMP-AMP synthase (cGAS) recognizes cytosolic viral DNA and triggers type I interferon (IFN-α/β) production to establish an antiviral state (Ablasser and Chen, 2019; Sun et al., 2013). In turn, viruses have evolved diverse strategies to evade host immunity. For instance, herpes simplex virus type 1 (HSV-1) targets the cGAS-STING pathway through UL41-mediated degradation of cGAS and UL46-mediated disruption of the STING-TBK1 complex (Su and Zheng, 2017; You et al., 2019). However, how PRV targets and counteracts cGAS-mediated innate immunity remains poorly understood.

Phase separation has emerged as a fundamental mechanism underlying the formation of membrane-less organelles, driven by the spontaneous concentration of proteins and nucleic acids under physiological conditions (Alberti et al., 2019; Banani et al., 2017). These dynamic condensates serve critical functions in diverse cellular processes, including gene transcription, signal transduction, and stress responses. Notably, several viruses have been reported to exploit phase separation to assemble replication compartments or subvert host immune responses (Chen and Cao, 2025; Heinrich et al., 2018; Nikolic et al., 2017). For instance, the rabies virus P protein antagonizes antiviral immunity through phase separation (Nevers et al., 2020), and human cytomegalovirus (HCMV) UL112-113 protein forms phase separation driven replication compartments to facilitate viral DNA replication (Caragliano et al., 2022). However, whether PRV utilizes phase separation to regulate host innate immunity remains unexplored.

Here, we performed PRV-encoded viral proteins bioinformatic analysis and identified the tegument protein pUL49 as the viral protein with the highest predicted propensity for phase separation. We show that pUL49 forms dynamic, 1,6-HEX-sensitive condensates in cells, colocalizes and interacts with cGAS, and suppresses poly(dA:dT)-induced IFN-*β* responses without significantly affecting cGAS transcript levels. Functionally, cGAS deficiency promotes PRV replication, whereas pUL49 depletion impairs viral replication. Collectively, these findings provide a mechanism by which PRV pUL49 exploits phase separation to dampen host antiviral defense and provide insights for therapeutic targeting of virus-host interactions.

## 2 Materials and methods

### 2.1 Cell culture and viruses

Human embryonic kidney 293T (HEK293T) and HeLa cell lines were obtained from the American Type Culture Collection (ATCC, Manassas, VA, United States). cGAS-deficient HeLa cells were stored in our lab. Cells were cultured in Dulbecco’s Modified Eagle Medium (DMEM, Gibco, Cat#11995500) supplemented with 10% (v/v) fetal bovine serum (FBS, ExCell Bio, Cat# FSP500) at 37 °C with 5% CO₂. PRV *Bartha-K61* was sourced from our laboratory stock, and viral titers were determined using the 50% tissue culture infectious dose (TCID₅₀) assay.

### 2.2 Plasmids and antibodies

The full-length coding sequence of PRV pUL49 was inserted into pEGFP-C3 and pCMV-Flag vectors to generate pEGFP-C3-pUL49 and pCMV-pUL49-Flag, respectively. The cGAS gene was inserted into the pCMV-mCherry and pCMV-Myc vectors for protein–protein interaction assays. The pRL-TK (Renilla) and pGL4-luc-IFN-β reporter plasmids were maintained in our laboratory. The following commercial antibodies were used: an anti-Myc antibody (Cat#562–5) was purchased from Medical & Biological Lab (MBL); anti-GFP (Cat#50430-2-AP), anti-Flag (Cat#20543-1-AP) and anti-Beta Actin (Cat#20536-1-AP) were obtained from the Proteintech Group.

### 2.3 Luciferase reporter assay

Cells were co-transfected with the IFN-β promoter-driven firefly luciferase reporter (IFN-β-Luc), pUL49 expression plasmid or empty vector control, and the pRL-TK Renilla luciferase plasmid as an internal control. After 24 h, cells were lysed, and luciferase activity was measured using the Dual-Luciferase Reporter Assay System (Beyotime, Cat#RG027) according to the manufacturer’s instructions. Relative luciferase activity was calculated by normalizing firefly luminescence to Renilla luminescence.

### 2.4 Quantitative real-time PCR (qRT-PCR)

Total RNA was extracted from cells using the RNeasy Mini Kit (Axygen, Cat#17921KD1) and reverse-transcribed into cDNA using the iScript cDNA Synthesis Kit (Vazyme, Cat#R323-01). qPCR was performed using the Hieff® qPCR SYBR Green Master Mix (11203ES03, Yeasen, Shanghai, China) and the Bio-Rad CFX96 Touch Real-Time Detection System. qPCR was performed using the following primers: PRV pUL49: forward 5′-CTCTCGCACCCGGACCA-3′, reverse 5′-CGTAGCCAT CGTAGCCGTAG-3′; PRV(gD): forward 5′-GGTTCAACGAGGGCCAGTACCG-3′, reverse 5′-GCG TCAGGAATCGCATCACGT-3′; cGAS: forward 5′-AGGAAGCAACTACGACTAAAGCC -3′, reverse 5′- CGATGTGAGAGAAGGATAGCCG -3′; ISG56: forward 5′- GGGAGTTATCCATT GATGACGATGA −3′, reverse 5′- GGTGTCTAGGAATTCAATCTGATCCAA −3′; β-actin: forward 5′-TGACGTGGACATCCGCAAAG-3′, reverse 5′-CTGGAAGGTGGACAGCGAGG-3′; IFN-β: forward 5′-CTCCTGGCTAATGTCT ATCA-3′, reverse 5′-GCAGAATCCTCCCATAATAT-3′.

### 2.5 Western blot

Cells were lysed on ice for 30 min in lysis buffer (20 mM Hepes pH 7.5, 150 mM NaCl, 1 mM EDTA, 1% (v/v) Triton X-100, 10% (v/v) glycerol, protease inhibitor cocktail). Lysates were subjected to immunoblotting with the indicated primary antibodies, followed by HRP-conjugated secondary antibodies. Protein signals were detected via enhanced chemiluminescence (ECL) substrate (Thermo Fisher Scientific) and captured using a Bio-Rad imaging system (Bio-Rad Laboratories, United States).

### 2.6 Co-immunoprecipitation (co-IP)

HEK293T cells were transfected with the indicated plasmids for 24–48 h, washed with ice-cold PBS, and lysed in IP lysis buffer [50 mM Tris–HCl pH 7.4, 150 mM NaCl, 1% (v/v) NP-40, protease inhibitor cocktail] on ice for 30 min. Lysates were centrifuged at 12,000 g for 15 min, and supernatants were incubated with the indicated antibodies and Protein A/G agarose beads overnight at 4 °C. After extensive washing, immunoprecipitates were eluted by boiling in SDS sample buffer and analyzed by immunoblotting.

### 2.7 RNA interference

shRNAs targeting pUL49 (shUL49) or scramble control shRNA were packaged into lentivirus in HEK293T cells. Sequences are listed below: shUL49-1, 5′-GACGACGATGACTACTACGGC-3′; shUL49-2, 5′-GGTGGTGCGCATCACCGTGTG-3′. The scramble control shRNA sequence is 5′-CAACAAGATGAAGAGCACCAA-3′. HeLa cells were then transduced with the lentiviral supernatants to establish stable knockdown cell lines. Transduced HeLa cells were seeded into 6-well plates and infected with PRV at an MOI of 1. Cells were harvested at 12 h post-infection for total RNA extraction. Knockdown efficiency and viral gene transcript levels were subsequently analyzed by RT-qPCR. Supernatants collected from parallel cultures were subjected to TCID₅₀ assay to determine viral titers.

### 2.8 Colocalization study using confocal microscopy

HeLa cells were seeded in confocal-specific dishes and co-transfected with GFP-pUL49 and mCherry-cGAS expression plasmids. At 24 h post-transfection, cells were fixed with 4% paraformaldehyde for 15 min at room temperature, washed three times with PBS, and stained with DAPI (Invitrogen) for 5 min to label nuclei. Images were acquired using a Leica TCS SP8 laser scanning confocal microscope (Leica Microsystems, Germany).

### 2.9 Fluorescence recovery after photobleaching (FRAP)

Cells were plated in 35 mm glass-bottom dishes and transfected with the pEGFP-C3-pUL49 plasmid, followed by 18 h of incubation. FRAP analysis was performed using a Nikon C2 confocal microscope (Nikon Corporation, Japan) equipped with a 37 °C chamber with 5% CO₂. After photobleaching a defined region of interest, time-lapse images were captured every 5 s for up to 150 s to monitor fluorescence recovery. Fluorescence intensity within the bleached region at each time point was quantified using ImageJ software.

### 2.10 Bioinformatic prediction

The amino acid sequences of PRV proteins were retrieved from the annotated complete genome of PRV strain XJ (GenBank accession: MW893682.1). We therefore restricted our analysis to 60 annotated proteins derived from the UL and US regions of the PRV XJ strain genome. Phase separation propensity of PRV-encoded proteins was predicted using the online servers FuzDrop^1^, PSPredictor^2^, and MolPhase^3^ with default parameters (Chu et al., 2022; Hatos et al., 2022; Liang et al., 2024). For FuzDrop, residues with P_DP_ ≥ 0.6 were classified as droplet-promoting regions; for MolPhase, residues with IDR ≥ 0.5 were defined as disordered regions with high phase separation propensity. Intrinsically disordered region (IDR) analysis of pUL49 was performed using IUPred3 and ANCHOR2 tools^4^ (Erdős et al., 2021). The pUL49-cGAS complex structure was predicted using AlphaFold3 by inputting the full-length amino acid sequences of both proteins, and the highest-confidence model was selected for subsequent analyses.

### 2.11 Virtual screening

Based on the AlphaFold3-predicted pUL49-cGAS complex structure, molecular docking was performed against 1,615 small-molecule ligands from the ZINC database using AutoDock Vina software. Screening criteria included a binding energy lower than −10.0 kcal/mol and binding localization within the pUL49-cGAS protein–protein interaction interface. Binding mode analysis of the final candidate compounds was visualized using PyMOL software.

### 2.12 Statistical analysis

Statistical analysis was performed using GraphPad Prism (version 10.0) software unless otherwise indicated. Student’s *t*-test was used to analyze the data. Values are presented as means ±standard deviation (SD). *p* < 0.05 was deemed to be significant.

## 3 Results

### 3.1 PRV pUL49 is predicted as a potent phase separating protein

**Figure 1:**
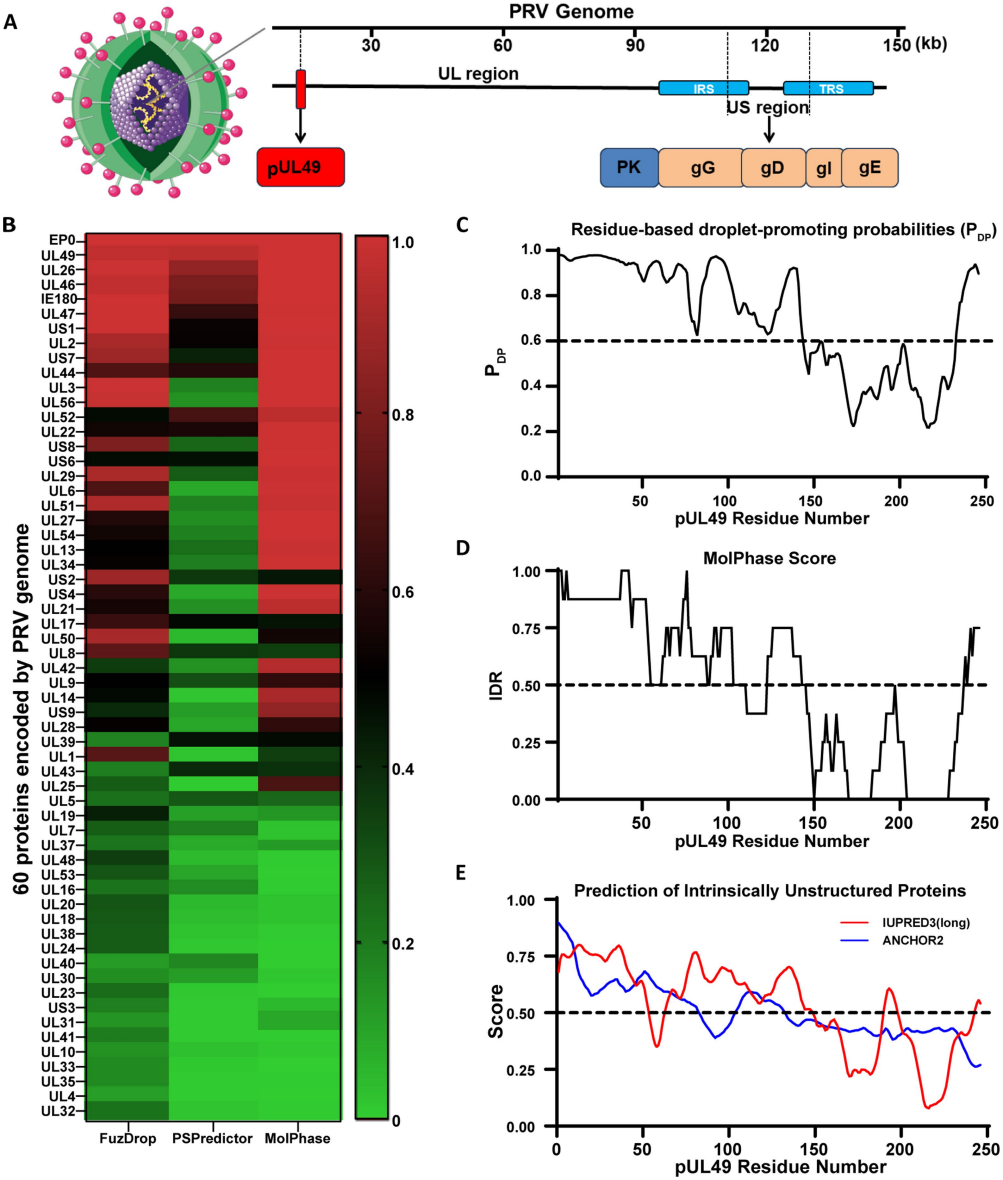
PRV UL49 is predicted as a potent phase separating protein. **(A)** PRV genome structure. **(B)** Phase separation prediction of 60 PRV-encoded proteins by FuzDrop, PSPredictor and MolPhase. FUS and GFP were positive and negative controls. **(C,D)** IDR prediction of pUL49 by FuzDrop **(C)** and MolPhase **(D)**. FuzDrop: P_DP_ ≥ 0.6; MolPhase: IDR ≥ 0.5. **(E)** IUPred3 (red) and ANCHOR2 (blue) analyses. Scores ≥0.5 indicate disordered regions (red) and potential protein–protein interaction sites (blue).

The PRV contains a typical herpesvirus genome consisting of unique long region (UL) and a unique short region (US) flanked by internal (IRS) and terminal (TRS) repeats (Figure 1A). To assess whether PRV-encoded proteins might exhibit phase separation potential, we analyzed 60 viral proteins using three prediction tools: FuzDrop, PSPredictor and MolPhase. Although EP0 has a high phase separation score, it is a transcriptional regulator and was not validated here; its phase separation function needs future study. Among the remaining proteins, the UL49-encoded tegument protein pUL49 exhibited the highest phase separation score (Figure 1B). pUL49, also known as VP22, is an evolutionarily conserved tegument protein of alphaherpesviruses, including HSV-1 and EHV-1. It is essential for viral replication and functions in virion assembly and intracellular trafficking (Wu et al., 2020). Sequence analysis further revealed that the N-terminal region of pUL49 (amino acids 1–150) is highly disordered, whereas the C terminus is comparatively ordered (Figures 1C,D). This disordered-ordered structural feature is characteristic of known phase separating proteins. Complementary analyses using IUPred3 and ANCHOR2 confirmed that residues 1–150 form a high-confidence IDR (Figure 1E). These analyses suggest that pUL49 possesses structural features consistent with phase separating proteins.

### 3.2 pUL49 forms dynamic condensates in cells

**Figure 2:**
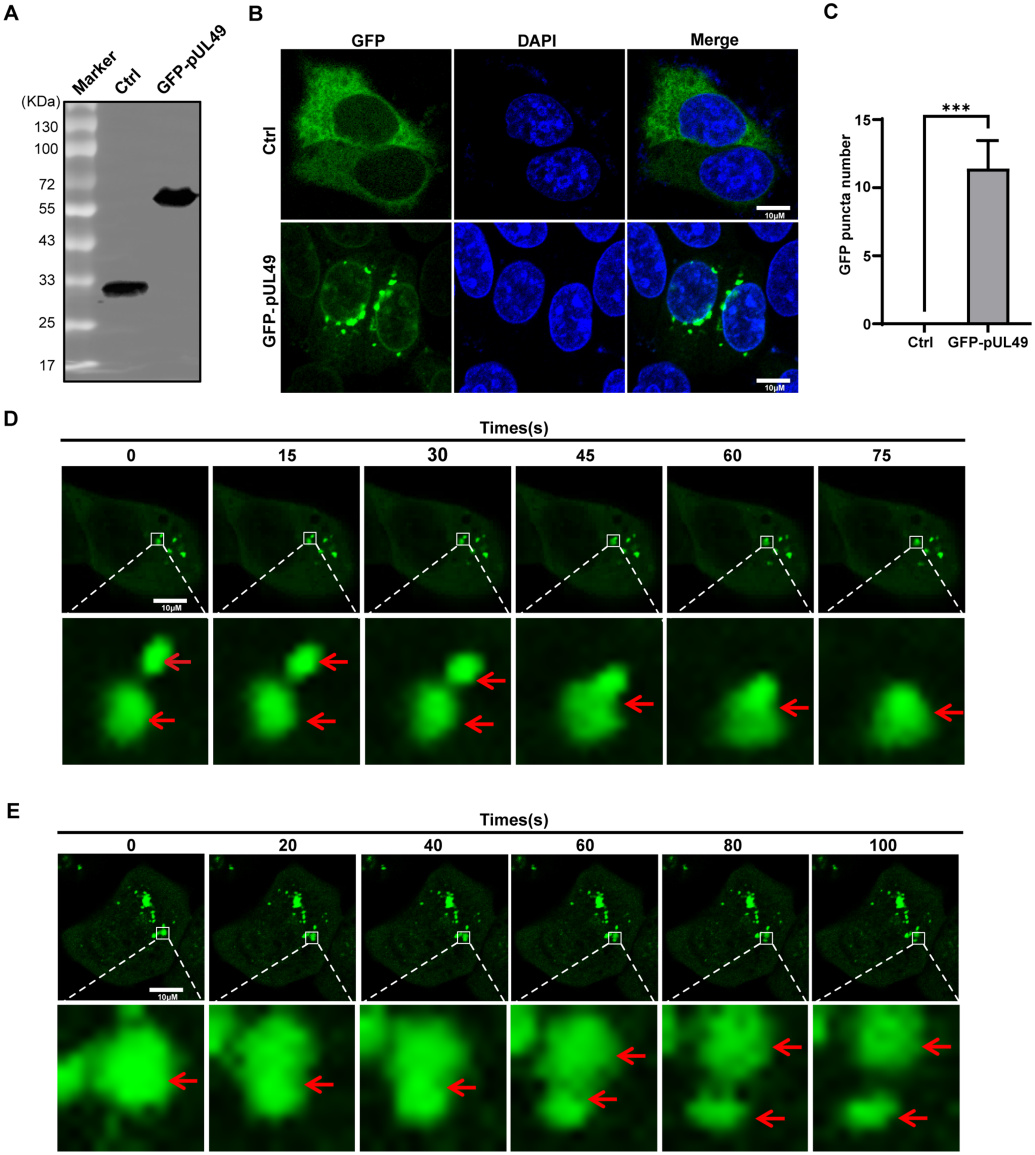
pUL49 forms dynamic condensates in cells. **(A)** Immunoblot validation of GFP-pUL49 expression in HeLa cells. GFP served as a control. **(B)** Confocal images of HeLa cells expressing GFP-pUL49 or GFP. Scale bar, 10 μm. **(C)** Quantification of puncta number in GFP-pUL49-transfected cells. Data are mean ± SD (*n* = 5). ***p < 0.001. **(D,E)** Time-lapse confocal imaging of GFP-pUL49 condensates. **(D)** Fusion event: two adjacent droplets fuse into a single larger droplet. **(E)** Fission event: a single condensate splits into two droplets. Arrowheads indicate fusion/fission sites. Scale bar, 10 μm.

To validate whether pUL49 possesses phase separation capacity, we constructed a GFP-tagged pUL49 expression plasmid (GFP-pUL49). Immunoblotting confirmed expression of the fusion protein in HeLa cells (Figure 2A). Confocal microscopy showed that GFP-pUL49 formed spherical puncta of varying sizes in the cytoplasm, whereas GFP alone displayed a diffuse distribution (Figures 2B,C), indicating that puncta formation is an intrinsic property of pUL49. Phase separation is the dynamic behavior of condensates, including fusion and fission. Live-cell imaging showed that adjacent small droplets gradually merged into a larger droplet (Figure 2D), whereas larger condensates split into two independent droplets (Figure 2E). These fusion and fission events are characteristic of liquid-like condensates and distinguish them from static aggregates. Collectively, these results demonstrate that pUL49 is an intrinsically phase separating protein capable of forming dynamic condensates with liquid-like properties in cells.

### 3.3 pUL49 condensates exhibit liquid-like properties

**Figure 3:**
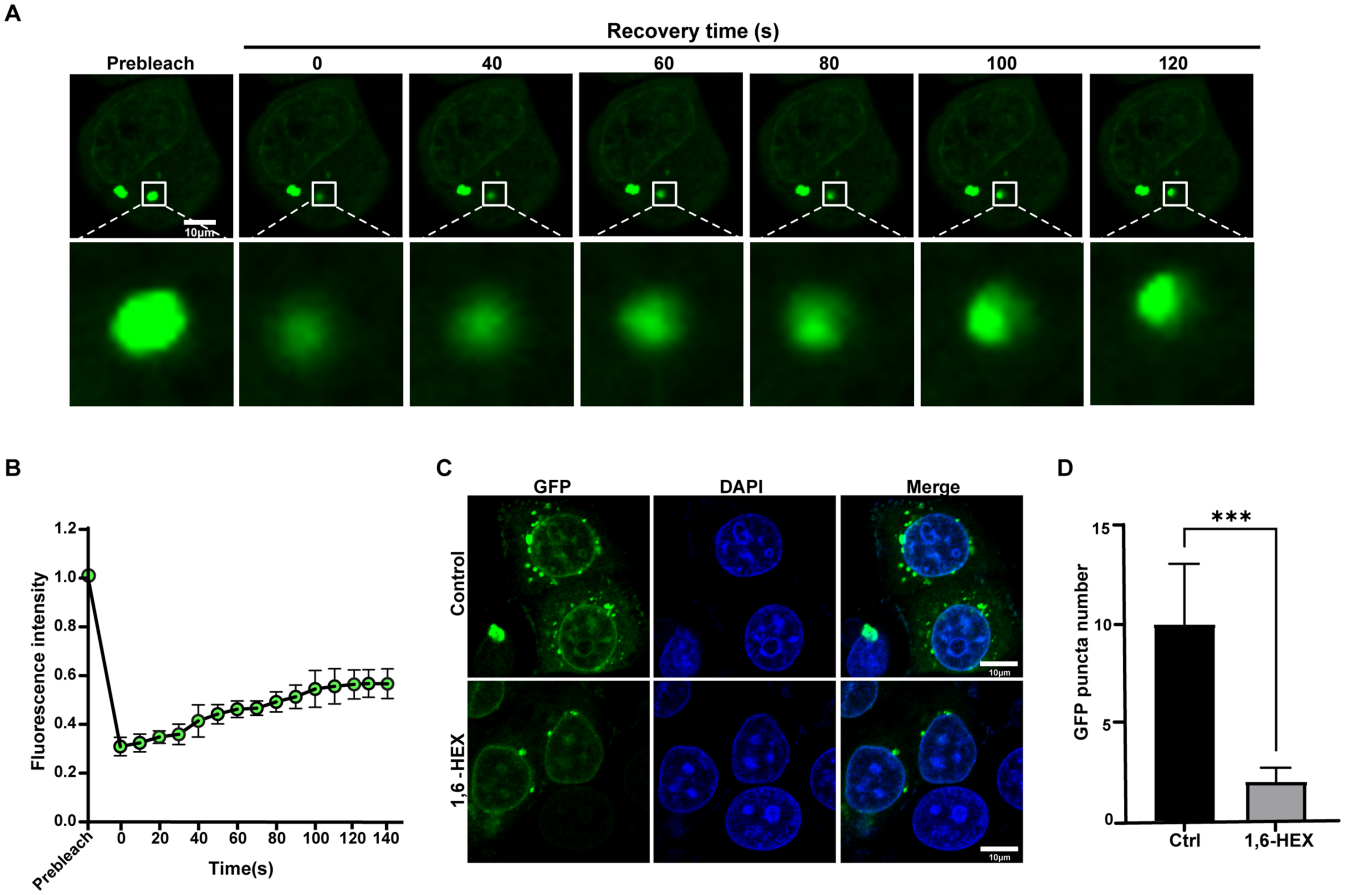
pUL49 condensates exhibit liquid-like properties. **(A)** FRAP analysis of GFP-pUL49 condensates in HeLa cells. A region of interest (ROI, white box) within a cytoplasmic droplet was photobleached, and fluorescence recovery was monitored over time. Scale bar, 10 μm. **(B)** Quantitative fluorescence recovery curve normalized to pre-bleach levels. Data are mean ± SD (*n* = 5). **(C)** HeLa cells expressing GFP-pUL49 were treated with 5% (w/v) 1,6-HEX for 10 min. Scale bar, 10 μm. **(D)** Quantification of droplet number per cell before and after 1,6-HEX treatment. Data are mean±SD (*n* = 5). ***p < 0.001.

Phase separation is the rapid exchange of components between condensates and their surroundings. To determine whether pUL49 condensates possess this property, we performed FRAP analysis. In HeLa cells expressing GFP-pUL49, a region of interest (ROI) within a cytoplasmic droplet was photobleached, and fluorescence recovery was monitored in real time. Fluorescence intensity recovered to approximately 60% of its initial level within 120 s (Figures 3A,B), indicating high internal molecular mobility and free diffusion of unbleached molecules into the bleached region. To further characterize pUL49 condensates, we treated GFP-pUL49-expressing cells with 5% 1,6-HEX (a phase separation inhibitor). Following treatment for 10 min, the number of GFP-pUL49 puncta was markedly reduced (Figures 3C,D). Together, these rapid FRAP recovery and the sensitivity to 1,6-HEX demonstrating the liquid-like properties of pUL49 condensates.

### 3.4 pUL49 interacts with cGAS and defines candidate targetable interface

**Figure 4:**
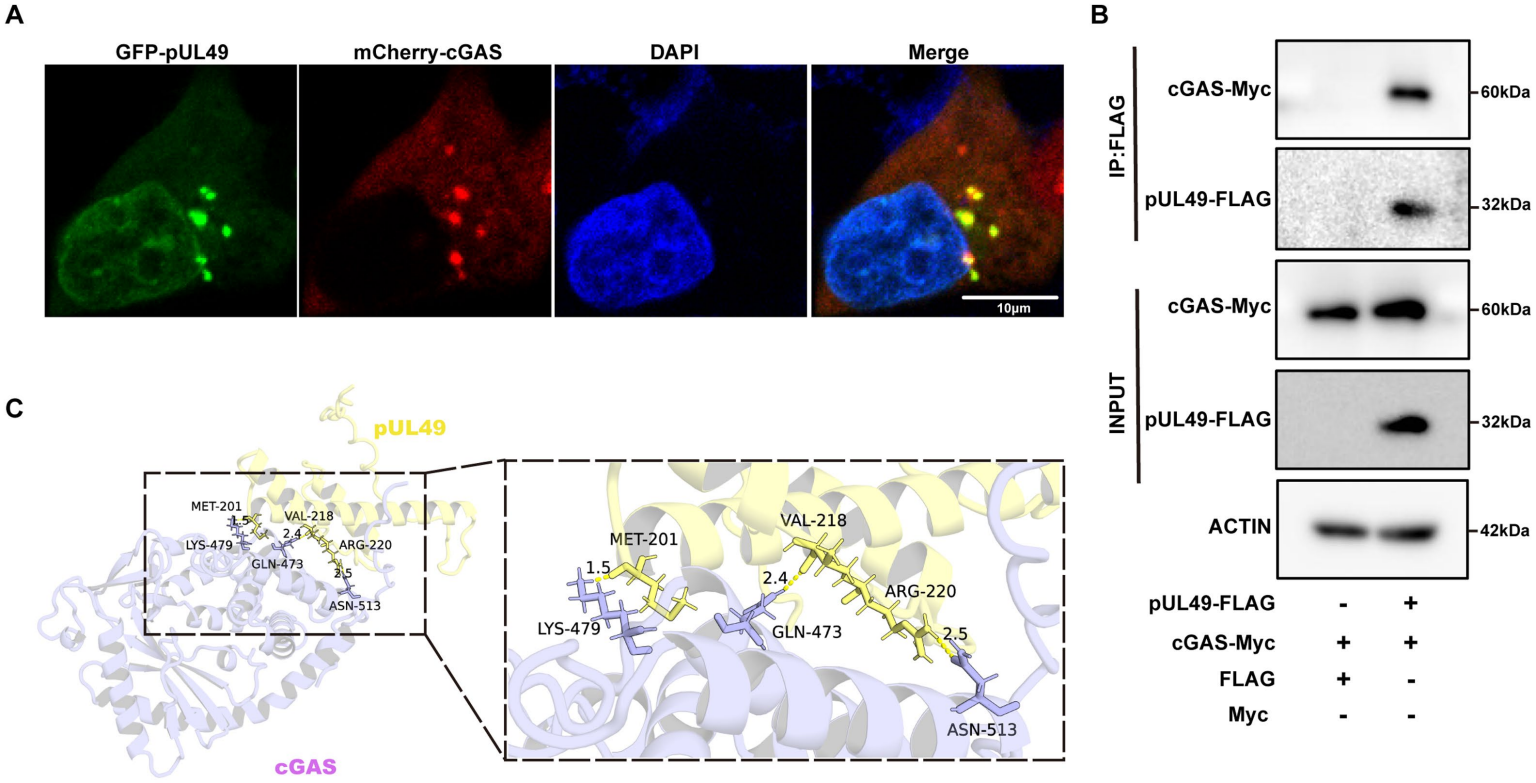
pUL49 interacts with cGAS. **(A)** Immunofluorescence colocalization of GFP-pUL49 (green) and mCherry-cGAS (red) in HeLa cells. Nuclei were stained with DAPI (blue). Merged images show yellow puncta. Scale bar, 10 μm. **(B)** Co-IP analysis of Flag-pUL49 and cGAS-Myc in HEK293T cells. Lysates were immunoprecipitated with anti-Flag antibody and immunoblotted with anti-Myc or anti-Flag antibodies. **(C)** AlphaFold3-predicted structural model of the pUL49-cGAS complex. pUL49 in yellow, cGAS in blue. The right panel shows the enlarged interface with key residues involved in hydrogen bonds or hydrophobic interactions.

Having established the phase separation properties of pUL49, we next investigated its biological functions. Given the central role of cGAS in antiviral immunity and reports that the HSV-1 homologous protein pUL49 targets cGAS (Eaglesham and Kranzusch, 2020; Huang et al., 2018), we hypothesized that PRV pUL49 could interact with cGAS via phase separation to modulate host innate immunity. Immunofluorescence colocalization showed that GFP-pUL49 and mCherry-cGAS extensively overlapped, forming distinct colocalized condensates in the perinuclear region (Figure 4A). Co-IP assays further showed that Flag-pUL49 specifically interacts with cGAS-Myc (Figure 4B). To gain structural insights, we predicted the pUL49-cGAS complex using AlphaFold3 Multimer. The model suggested a potential interface between the N-terminal disordered region of pUL49 and the C-terminal domain of cGAS, involving both putative hydrogen bonds and hydrophobic contacts, including a predicted interaction between pUL49 Val218 and cGAS Gln473(Figure 4C). Sequence alignment of different PRV strains confirmed that these residues are highly conserved. Collectively, these results indicate that pUL49 colocalizes and interacts with cGAS within phase separated condensates, raising a possibility that this interaction occurs in the context of pUL49 containing cGAS condensates.

Next, we used the modeled interface as the template for structure-guided virtual screening. From a library of 1,615 ZINC compounds, 20 top-ranked molecules were selected for further analysis, 8 candidates were predicted to occupy the putative pUL49-cGAS interface. Notably, several of these compounds, including conivaptan and venetoclax, are FDA-approved drugs (Supplementary Figure S1). Although these findings remain predictive and require experimental validation, we identify the pUL49-cGAS interface as a candidate site for therapeutic interrogation.

### 3.5 pUL49 suppresses cGAS mediated type I interferon signaling

**Figure 5:**
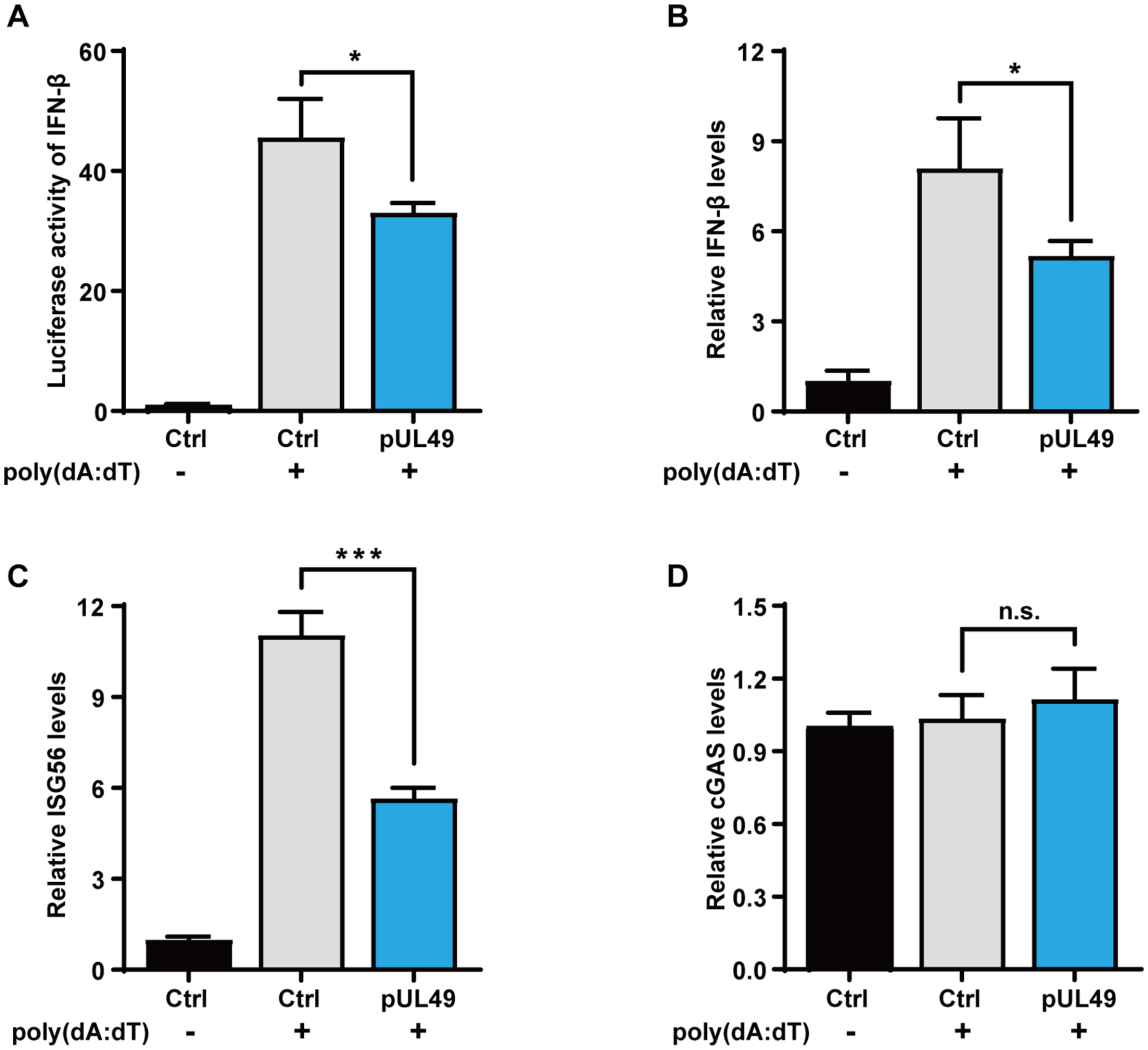
pUL49 suppresses cGAS mediated type I interferon signaling. **(A)** Dual-luciferase reporter assay of IFN-β promoter activity. HEK293T cells were transfected with IFN-β-Luc and pUL49 or empty vector for 24 h, followed by transfection with poly(dA:dT) (2 μg/mL) for 6 h. Data are mean ± SD (*n* = 3). *p < 0.05. **(B)** qRT-PCR analysis of IFN-β mRNA levels. Cells were transfected with pUL49 or empty vector for 24 h, followed by transfection with poly(dA:dT) (2 μg/mL) for 6 h. Data are mean ± SD (*n* = 3). *p < 0.05. **(C)** qRT-PCR analysis of ISG56 mRNA levels. Cells were transfected with pUL49 or empty vector for 24 h, followed by transfection with poly(dA:dT) (2 μg/mL) for 12 h. Data are mean ± SD (*n* = 3). ***p < 0.001. **(D)** qRT-PCR analysis of cGAS mRNA levels. Cells were treated as in **(B)**. Data are mean ± SD (*n* = 3). ns, not significant.

To determine the functional significance of this interaction, we next assessed its impact on cGAS downstream signaling. Using a dual-luciferase reporter assay, we found that pUL49 overexpression significantly suppressed poly(dA:dT)-induced IFN-β promoter activation (Figure 5A). Consistently, qRT-PCR analysis showed that pUL49 overexpression markedly reduced the mRNA levels of IFN-β and its downstream gene ISG56 upon poly(dA:dT) stimulation (Figures 5B,C). Notably, cGAS mRNA levels were not significantly altered by pUL49 overexpression (Figure 5D). Together, these results demonstrate that pUL49 specifically suppresses cGAS-mediated type I interferon responses.

### 3.6 pUL49 promotes PRV replication by antagonizing cGAS

**Figure 6:**
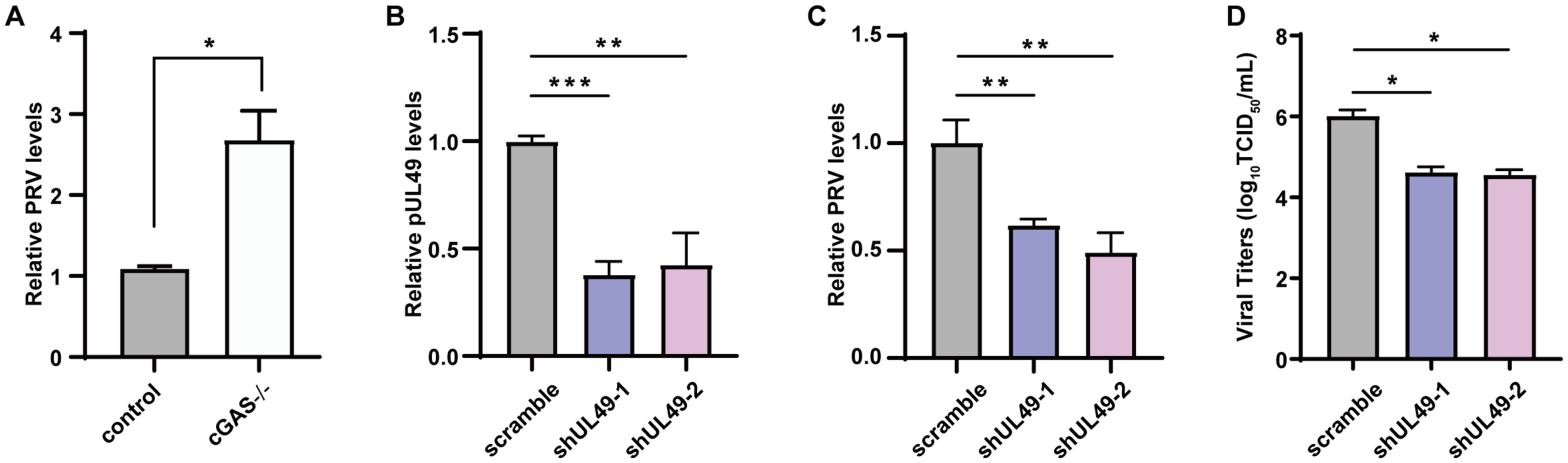
pUL49 promotes PRV replication by antagonizing cGAS. **(A)** Effect of cGAS knockout on PRV replication. WT and cGAS-KO HeLa cells were infected with PRV (MOI = 0.1). Viral mRNA levels were quantified by RT-qPCR at 24 hpi. Data are mean ± SD (*n* = 3). *p < 0.05. **(B)** Validation of pUL49 knockdown efficiency. HeLa cells transduced with Scramble or shUL49 were infected with PRV (MOI = 1.0). pUL49 mRNA levels were quantified by RT-qPCR at 12 hpi. Data are mean ± SD (*n* = 3). **p < 0.01, ***p < 0.001. **(C)** Effect of pUL49 knockdown on viral gene expression. Cells were treated as in **(B)**, and viral mRNA levels were quantified by RT-qPCR at 12 hpi. Data are mean ± SD (*n* = 3). **p < 0.01. **(D)** The titers of PRV infectious progeny virions in pUL49 knockdown HeLa cells were detected via TCID₅₀ assay. Data are mean ± SD (*n* = 3). *p < 0.05.

To assess the cGAS-mediated interferon signaling in PRV infection, we examined viral replication in cGAS-knockout (cGAS-KO) HeLa cells generated by CRISPR-Cas9. Viral mRNA levels were significantly higher in cGAS-KO cells than in wild-type controls (Figure 6A), indicating that cGAS restricts PRV infection. Conversely, to evaluate the role of pUL49 in viral replication, we knocked down pUL49 expression using specific shRNA. Efficient knockdown was confirmed by RT-qPCR (Figure 6B), and under these conditions viral mRNA levels were markedly reduced (Figure 6C), demonstrating pUL49 positively regulates PRV replication. To further verify above findings, progeny viral titers determined by TCID₅₀ assay were consistent with the alteration of viral mRNA levels (Figure 6D). Taken together, pUL49 promotes PRV replication via inhibition of cGAS-mediated antiviral signaling.

## 4 Discussion

Phase separation is increasingly implicated in virus and host interactions (Li et al., 2022; Yang et al., 2023). However, previous work has focused on replication compartment assembly, while its role in immune evasion during PRV infection remains unclear. In this study, we identify pUL49 as a condensate-forming tegument protein that suppresses cGAS-dependent antiviral signaling and promotes PRV replication.

In herpesviruses, phase separation has been considered as a mechanism for organizing viral replication microenvironments (Zhou et al., 2023). In HCMV, UL112-113 undergoes phase separation at viral genomes and recruits the viral polymerase factor UL44 (Caragliano et al., 2022), which promotes formation of a pro-replicative nuclear compartment and concentrates replication factors locally. Notably, HSV-1 VP22 disrupts cGAS-DNA phase separation to evade host innate immunity (Xu et al., 2021). PRV VP22 blocks cGAS condensate assembly and cGAMP production by regulating cytoplasmic translocation of DDX21 (Liu et al., 2025). By contrast, our data support a role for PRV pUL49 condensates in innate immune suppression via antagonizing cGAS function. Such functionality suggests that pUL49 may participate in both viral compartment formation and host immune antagonism. Whether PRV pUL49 similarly interferes with DNA-triggered cGAS phase separation remains to be determined. Future work will focus on exploring whether pUL49 condensates recruit distinct host factors to regulate innate immune signaling.

Our findings further suggest PRV pUL49 may represent a condensate-associated cGAS antagonist. In VZV, the tegument protein ORF9 act as a cGAS antagonist by binding both cGAS and DNA, undergoing phase separation with DNA, and inhibiting cGAMP production (Hertzog et al., 2022). In SARS-CoV-2, the nucleocapsid protein suppresses cGAS-mediated signaling through DNA-induced phase separation and disrupt the cGAS-G3BP1 pathway (Cai et al., 2023). Our data suggest that pUL49-mediated condensate formation contributes to suppression of cGAS-dependent antiviral signaling during PRV infection. The stage at which pUL49 exerts this suppressive effect remains to be explored.

Recent studies suggest emerging PRV variants have zoonotic potentials, with sporadic human spillover cases causing severe and often fatal infections in humans (Ai et al., 2018; Liu et al., 2021; Wang et al., 2026). How PRV gains fitness across host barriers remains poorly understood. Therefore, inhibition of cGAS-dependent antiviral restriction by pUL49 and other tegument proteins may enhance viral fitness. Given its critical role in promoting efficient PRV replication, pUL49 represents a promising target for antiviral intervention. Our virtual screening analysis lays a foundation for the subsequent development of small-molecule inhibitors to block PRV replication. While these *in silico* predictions are valuable, they require experimental validation. Specifically, the predicted pUL49-cGAS interface needs to be verified by site-directed mutagenesis, and the antiviral activity of the identified compounds remains to be assessed.

In conclusion, this study demonstrates that the PRV tegument protein pUL49 forms biomolecular condensates, inhibits cGAS-dependent antiviral signaling, and promotes viral replication. These findings provide mechanistic insights into how viral proteins remodel the intracellular antiviral milieu to support viral infection.

## Data Availability

The original contributions presented in the study are included in the article/Supplementary material, further inquiries can be directed to the corresponding authors.

## Footnotes

1
https://fuzdrop.bio.unipd.it/

2
http://www.pkumdl.cn:8000/PSPredictor/

3
https://molphase.sbs.ntu.edu.sg/

4
https://iupred3.elte.hu/
