# Supplementary material for: Pseudorabies virus tegument protein pUL49 antagonizes cyclic GMP-AMP synthase through phase separation to promote viral replication
Source: Front Microbiol. 2026 Jul 2;17:1873742. doi: 10.3389/fmicb.2026.1873742 (PMC13372753; doi:10.3389/fmicb.2026.1873742)
Supplement: Supplementary file 1 [file Table_1.docx]

Supplementary Material

**Supplementary Figure 1.** **Virtual screening identifies candidate compounds targeting the pUL49-cGAS interface**


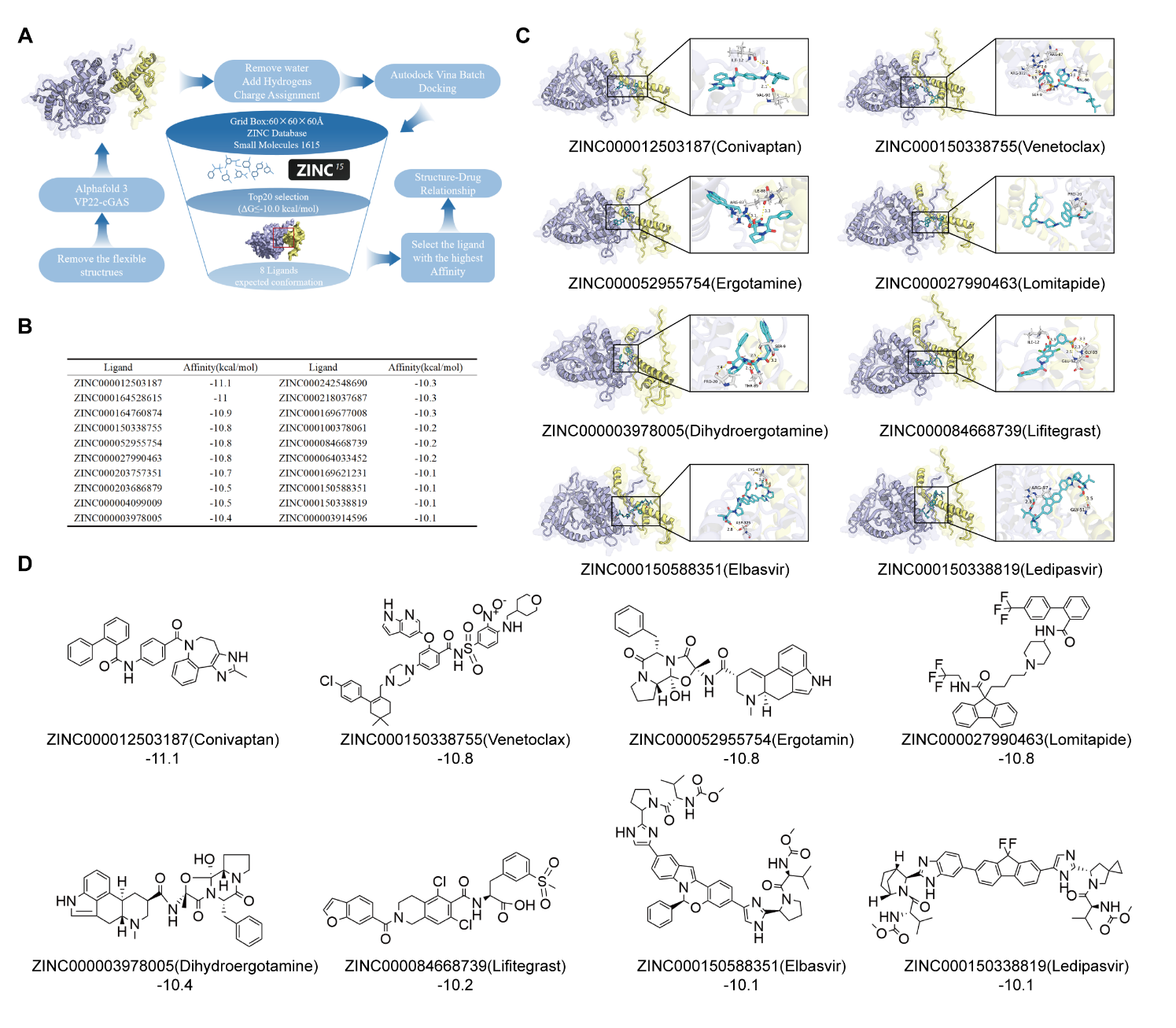


(A) Virtual screening workflow using AlphaFold3-predicted pUL49-cGAS structure against 1,615 compounds from ZINC database. (B) Binding energy distribution of top 20 candidates (all < -10.0 kcal/mol). (C) Spatial filtering strategy: after energy-based selection, compounds were filtered by binding position; only those docking into the pUL49-cGAS interface were retained. (D) Chemical structures and binding energies of eight final candidates. Several are FDA-approved drugs (e.g., conivaptan, venetoclax, ergotamine), suggesting repurposing potential.
